# Feasibility, Acceptability, Satisfaction, and Challenges of an mHealth App (e-ASCov) for Community-Based COVID-19 Screening by Community Health Workers in Rwanda: Mixed Methods Study

**DOI:** 10.2196/50745

**Published:** 2024-10-14

**Authors:** Abdou Y Omorou, Pacifique Ndishimye, Bruno Hoen, Léon Mutesa, Prosper Karame, Ladislas Nshimiyimana, Simon Galmiche, Hassan Mugabo, Janvier Murayire, Muco Mugisha, Marie Michele Umulisa, Yvonne Delphine Nsaba Uwera, Clarisse Musanagabanwa, Noella Bigirimana, Sabin Nsanzimana, Francis Guillemin, Jean Paul Rwabihama

**Affiliations:** 1 UMR 1319 INSPIIRE, INSERM, Université de Lorraine, Vandoeuvre-Lès-Nancy, France; 2 CHRU Nancy, INSERM, Université de Lorraine, CIC Clinical Epidemiology, Vandoeuvre-Lès-Nancy, France; 3 Laboratory of Emerging and Infectious Diseases, School of Medicine, Dalhousie University, Halifax, NS, Canada; 4 Research, Innovation and Data Science Division, Rwanda Biomedical Centre, Kigali, Rwanda; 5 Direction de la recherche médicale, Institut Pasteur, Paris, France; 6 Centre for Human Genetics, College of Medicine and Health Sciences, University of Rwanda, Kigali, Rwanda; 7 Emerging Diseases Epidemiology Unit, Institut Pasteur, Université Paris Cité, Paris, France; 8 Department of Internal Medicine, College of Medicine and Health Sciences, University of Rwanda, Kigali, Rwanda; 9 Rinda Ubuzima, Kigali, Rwanda; 10 Midwifery Department, School of Nursing and Midwifery, College of Medicine and Health Sciences, University of Rwanda, Kigali, Rwanda; 11 Ministry of Health Rwanda, Kigali, Rwanda

**Keywords:** community health workers, COVID-19 screening tool, COVID-19, SARS-CoV-2, screening, acceptability, feasibility, satisfaction, community based, LMIC, Africa, challenges, barriers, smartphone, proof-of-concept, mHealth, mobile health, apps, COVID-19 screening

## Abstract

**Background:**

Although at the base of the pyramid-shaped organization of the Rwandan health system, community health workers (CHWs) are central to the community-based management of disease outbreaks.

**Objective:**

This mixed methods study aimed to explore the feasibility, acceptability, satisfaction, and challenges of a mobile health (mHealth) tool for community-based COVID-19 screening in Rwanda.

**Methods:**

Two urban (Gasabo and Nyarugenge) and 2 rural (Rusizi and Kirehe) districts in Rwanda participated in the project (smartphone app for COVID-19 screening). A mixed methods approach was used to inform the feasibility (awareness and expectation), acceptability (use and perceived benefits), satisfaction, and challenges of the mHealth intervention. At the end of the project, CHWs were asked to complete a quantitative questionnaire on the use of and satisfaction with the app. Then, in-depth interviews and focus group discussions were organized with CHWs. A content analysis was performed on the transcripts.

**Results:**

Overall, 383 CHWs were recruited and trained; 378 CHWs participated in the study. The mean age of CHWs was 36.7 (SD 6.6) to 45.3 (SD 9.9) years and most were women (237/378, 62.7%). More than 7000 people were registered with the use of the app and 20% were referred to a local COVID-19 testing facility. According to CHW reporting, the median number of people screened by each CHW ranged from 152 (IQR 70-276) for Nyarugenge to 24 (IQR 16-90) for Rusizi. COVID-19 positivity rates were higher in urban than rural districts: more than half of the CHWs in Gasabo reported a confirmed positive case versus only 2.4% for Kirehe and 15.4% for Rusizi. Despite the app being a novel tool, CHWs were well aware of the use of such a tool and had appropriate expectations. Acceptability and satisfaction were very high, with differences between urban and rural districts. Satisfaction was higher in Nyarugenge (72.8/100) and Gasabo (80.7/100) than in Kirehe (61.6/100) and Rusizi (64.5/100). More than 80% of the CHWs were willing to continue using the e-ASCov app, with the exception of CHWs in Kirehe (56.7%). The app was perceived as a tool to generate information on COVID-19, inform on the status of the pandemic, and help curb the spread of the pandemic in Rwanda. CHWs were satisfied with the app at all stages of its implementation in their districts.

**Conclusions:**

In this proof-of-concept study, a smartphone app for screening COVID-19 was useful as an mHealth tool to be used by CHWs, with the potential to increase health system efficiency in an epidemic context. The context should be analyzed for generalization on a country-wide scale, both in case of an epidemic and to take into account certain conditions at the community level. Information is needed on the conditions of generalization and transferability of this type of app to other health conditions so that CHWs can be given their full place in a pyramidal health system.

## Introduction

Since December 2019, the appearance of a new strain of coronavirus (SARS-Cov-2) has been at the origin of the current COVID-19 pandemic [1]. In Africa, the first case of COVID-19 was reported on February 14, 2020, in Egypt and on February 27, 2020, in Nigeria, sub-Saharan Africa. With lockdowns, curfews, closures of public places and businesses, and the suspension of international flights, the continent’s public health leaders tried to contain the epidemic. Rwanda was greatly affected by the COVID-19 pandemic, with initially a majority of imported cases.

The Rwandan government, through the Ministry of Health and the Rwanda Biomedical Center, a national agency for developing and implementing public health programs, developed a nationwide plan to deal with the COVID-19 epidemic [2]. This action plan aimed to improve Rwanda’s ability to prevent, rapidly diagnose, and effectively respond to the COVID-19 epidemic and was structured around prevention and management. Prevention activities were mainly based on epidemiological surveillance, human resources, patient transfer, hygiene reinforcement, social mobilization and coordination, and monitoring activities [3]. A multisectoral rapid response team was activated at central and local levels to respond to the pandemic [4].

The pyramidal organization of the Rwandan health system gives an important place to community health workers (CHWs) at the base of the pyramid. Rwanda has an important network of CHWs throughout the country. Their role is essential in the field of primary health care. These CHWs are the essential link between the population and the health care system at the community level. The activation of this network and the provision of simple and ergonomic tools for epidemic surveillance were seen as an effective and efficient way to combat this epidemic.

The experience of the Ebola epidemic in 3 sub-Saharan African countries (Guinea, Liberia, and Sierra Leone) showed that CHWs played a key role in controlling the epidemic, but it also highlighted the need to involve CHWs very early in the epidemic response to ensure optimal effectiveness [5,6]. Some health actors believed that with this COVID-19 pandemic, CHWs were more than ever indispensable in addressing the disease [7]. However, formal evidence was needed. Although long-term solutions to the pandemic would be based on the rollout of vaccines and effective treatments, digital technologies remained crucial in terms of detection during the pandemic.

In light of its strategic objectives to leverage opportunities and innovations of communication technology and essentially drive the transformation of Rwanda to a knowledge-based economy, Rwanda has embraced innovative technologies as part of the COVID-19 early case detection response [8]. In this regard, the Rwanda Biomedical Center and its partners (CIC de Nancy, and Institut Pasteur, Paris, France) launched the e-ASCov project (Utilisation des outils numériques par les agents de santé communautaire dans la stratégie d’endiguement et d’atténuation de l’épidémie de Covid-19 au Rwanda–une recherche-action). This project aimed to assess the involvement of CHWs and the use of digital tools (mobile health [mHealth] smartphone app) in managing the COVID-19 pandemic in Rwanda in 2020.

This study aimed to assess the feasibility (awareness and expectations), acceptability (use and perceived benefits), satisfaction, and challenges of an mHealth tool for community-based COVID-19 screening in Rwanda.

## Methods

### The e-ASCov Project

The e-ASCov project is an action-research project that enrolled, trained, and equipped CHWs in 4 administrative districts in Rwanda: 2 urban (Nyarugenge and Gasabo) and 2 rural (Rusizi and Kirehe). CHWs were equipped with a smartphone app (e-ASCov app) to detect and triage suspected COVID-19 cases at the community level and to propose referral to the health care system for appropriate management. A total of 400 CHWs were selected (100 per district) based on 2 criteria: age <50 years and availability and proficiency in the use of a smartphone. The project was carried out from November 2020 to December 2021. Phase 1 of the project emphasized training and equipping CHWs with the e-ASCov app, and facilitating them to use the app in their day-to-day activities. Phase 2 emphasized an evaluation of the implementation of the e-ASCov app for the management of the COVID-19 pandemic. A parallel mixed (quantitative and qualitative) method was used to highlight the implementation of the e-ASCov app for managing COVID-19 at the community level.

### Ethical Considerations

This project was approved by the Rwanda National Ethics Committee (number 752/RNEC/2020) as well as the Rwanda CHW Technical Working Group. The target population consisted of CHWs trained in the use of digital tools (e-ASCov app) for managing the COVID-19 pandemic. Informed consent was required from all participants before using the app, and they had the ability to opt out without any consequence. All data collected were anonymized and without the possibility of participant identification. No compensation was given to participants.

### Data Collection

#### Quantitative Data

All CHWs were asked to complete a questionnaire at the end of the intervention (use of the e-ASCov app by CHWs). The questionnaire covered sociodemographic characteristics (sex, age, educational attainment, occupation, marital status, etc), the description of the intervention (number of persons screened, persons referred, confirmed cases), and satisfaction with the e-ASCov app. Satisfaction was measured on a 5-point Likert scale (very satisfied, satisfied, undecided/neutral, unsatisfied, very unsatisfied) that addressed the following dimensions: quality of training, availability of equipment, internet connectivity, simplicity/use of the tool, time needed to complete one record, getting technical assistance, delivery of service to clients, and continued use of the tool.

#### Qualitative Data

In-depth interviews (IDIs) and focus group discussions (FGDs) were conducted: 2 FGDs with CHWs and 2 with clients of CHWs per district (4 FGDs per district). Each FGD consisted of 8 participants, for a total of 64 CHWs and 64 clients. For the IDIs, a purposive sampling strategy was used to recruit key members of staff (who were not directly involved in using the app in the field) from the following 3 categories: COVID-19 taskforce members, data managers at central and district levels, and CHW supervisors at central and district levels. Qualitative data management and analysis involved verbatim transcription of interviews, translation, and thematic analysis. Interviews were conducted in Kinyarwanda, transcribed using Microsoft Word, and translated into English. The transcripts were cross-checked for quality by 2 analysts who compared audio records and scripts in Kinyarwanda, then with the English translation, considering what was directly said by the respondent. Each individual transcript was used for analysis.

### Data Analysis

Qualitative and quantitative data were analyzed to address the question of feasibility (awareness and expectations), acceptability (use and perceived benefits), satisfaction, and challenges.

#### Quantitative Data

Sociodemographic characteristics are described with mean and SD for continuous and number (percentage) for categorical variables. We compared characteristics according to districts of participants by Student *t* test or chi-square test as applicable. Then we compared the number of persons screened with or without e-ASCov, persons referred to the district hospital, and confirmed cases by the district of the CHWs. A satisfaction score was calculated and normalized on a scale from 0 to 100 (highest satisfaction) for each dimension, and a global satisfaction score was calculated.

#### Qualitative Data

An inductive thematic analysis approach was used to analyze qualitative data in the following 6 areas: familiarization, coding (each code described the idea or the feeling expressed in the specific part of the text under the topic of discussion), generating themes and subthemes (ideas and patterns that came up repeatedly), reviewing themes, defining and naming themes, and writing up [9]. The analysis was conducted with Dedoose software (version 9.0.18), a web-based cross-platform for qualitative analysis and mixed methods research.

## Results

### Sample Characteristics

Of the 400 CWHs recruited, 378 CWHs completed the quantitative questionnaires (Gasabo region: n=93; Kirehe: n=97; Nyarugenge: n=90; Rusizi: n=98). The mean age of CHWs ranged from 36.7 (SD 6.6) to 45.3 (SD 9.9) years (Table 1). CHWs were mostly women (237/378, 62.7%) with primary or secondary educational attainment. CHWs were mostly unemployed in the districts of Gasabo (52/93, 55.9%) and Kirehe (71/97, 73.2%), whereas those from the 2 other districts were mostly employed (Nyarugenge: 53/90, 58.9%; Rusizi: 79/98, 80.6%). Most were experienced CHWs, with a mean number of years of experience of 7.3 to 10.1.

The IDIs involved 19 respondents: 5 CHW supervisors (2 at the central level and 3 at the district level), 5 CHWs from the COVID-19 taskforce (2 at the central level, 3 at the district level), 3 data managers from the district hospitals, 2 director generals of the hospitals, and 4 clinical directors at the district level. The FGDs involved 64 CHWs from all 4 districts included in this study.

**Table 1.** Sociodemographic characteristics of community health workers according to district in Rwanda.

Characteristics		Gasabo (n=93, 24.6%)	Kirehe (n=97, 25.7%)	Nyarugenge (n=90, 23.8%)	Rusizi (n=98, 25.9%)	*P* value^a^
**Age (years)**						<.001
	Participants, n (%)	93 (100.0)	97 (100.0)	90 (100.0)	98 (100.0)	
	Mean (SD)	36.7 (6.6)	43.6 (7.1)	40.5 (7.6)	45.3 (9.9)	
**Sex, n (%)**						.01
	Male	27 (29.0)	45(46.4)	26 (28.9)	43 (43.9)	
	Female	66 (71.0)	52 (53.6)	64 (71.1)	55 (56.1)	
**Type of district, n (%)**						<.001
	Rural	0 (0.0)	97 (100.0)	0 (0.0)	98 (100.0)	
	Urban	93 (100.0)	0 (0.0)	90 (100.0)	0 (0.0)	
**Educational attainment, n (%)**						<.001
	Primary	15 (16.1)	63 (64.9)	20 (22.2)	63 (64.3)	
	Secondary	52 (55.9)	28 (28.9)	54 (60.0)	26 (26.5)	
	TVET^b^	0 (0.0)	3 (3.1)	2 (2.2)	5 (5.1)	
	University	26 (28.0)	3 (3.1)	14 (15.6)	4 (4.1)	
**Occupation, n (%)**						<.001
	Employed	41 (44.1)	26 (26.8)	53 (58.9)	79 (80.6)	
	Unemployed	52 (55.9)	71 (73.2)	37 (41.1)	19 (19.4)	
**Marital status, n (%)**						<.001
	Divorced/separated	2 (2.2)	2 (2.1)	2 (2.2)	2 (2.0)	
	Married/living with the partner	64 (68.8)	90 (92.8)	62 (68.9)	86 (87.8)	
	Single/never married	26 (28.0)	2 (2.1)	15 (16.7)	3 (3.1)	
	Widowed	1 (1.1)	3 (3.1)	11 (12.2)	7 (7.1)	
**Function, n (%)**						<.001
	MCHC^c^	20 (21.5)	13 (13.4)	30 (33.3)	11 (11.2)	
	Binome	56 (60.2)	59 (60.8)	52 (57.8)	76 (77.6)	
	CEHO^d^	10 (10.8)	2 (2.1)	7 (7.8)	3 (3.1)	
	Prevention	7 (7.5)	23 (23.7)	1 (1.1)	8 (8.2)	
**Years of experience**						.002
	Participants, n (%)	93 (100.0)	97 (100.0)	90 (100.0)	98 (100.0)	
	Mean (SD)	7.3 (4.5)	9.6 (5.7)	8.6 (4.8)	10.1 (6.1)	

a *P* value for *χ*2 test for qualitative variables or Student *t* test for quantitative variables.

b TVET: technical vocation education training.

c MCHC: maternal and child health coordinator.

d CEHO: community and environmental health officer.

### Awareness of and Expectations for the e-ASCov App

Most respondents were aware of the e-ASCov app and believed that it was used to provide information on COVID-19. These respondents were located predominantly in urban districts (Nyarugenge and Gasabo).

e-ASCov is the application which is used by the community health workers to help people who are suspected of COVID-19 symptoms

[COVID-19 Taskforce, central level]

The CHWs, especially the respondents with the opportunity to use the app, considered it a tool to generate information on COVID-19.

Those who gave us training, they explained to us how to write a report on COVID-19 using forms for identified clients; after filling the forms, we could take them to the health center. That is how we worked

[CHW, Kirehe District, FGD]

About knowledge on e-ASCOv, I can tell you that we do not have any because the only training we received was on how to use the forms.

[CHW, Kirehe District]

The respondents expected the app to help inform on the status of the pandemic and help with community outreach on COVID-19. Even though some CHWs were not aware of the app and did not have any expectations, a number of them were expecting the e-ASCov app to help curb the spread of COVID-19. They also expected it to support an extension of the service to home-based care, provide support to cases, and improve CHWs knowledge and the app to be used by all CHWs.

What we have expected and still expect with current results is that the use of technology has impacted well in curbing the spread of COVID-19

[COVID-19 Taskforce, district level]

What we all expect is that the app contributes [to] the continuous fight against COVID-19, like getting information on time

[CHW supervisor, central level]

As I said, what we were expecting is to find out the status of the pandemic, and other measures that could be taken to prevent it because there is no other way to use it without the technology

[district director of health]

The CHWs’ expectations were mostly that the app would provide an update on the status of the pandemic in their area. They thought that the detection of COVID-19 cases would lead to a decrease in the magnitude of the pandemic. In addition, some CHWs emphasized the app’s contribution to early diagnosis and treatment and the opportunity to screen other health commodities as in their routine community-based health services. Some clients also considered that screening with the technology would reduce the burden of COVID-19.

What we expected was the results of using this application in helping Rwandans during the pandemic. We can say, like follow-up of the infected people before they get complications for them to receive early medical management, for the health system at central level to get information on time and all the people with the disease to be managed on time. I think that was what we expected and the results have been seen

[CHW, Gasabo District, FDG]

We expected to provide early and quick information if someone was having the signs and symptoms for the healthcare providers to provide early and timely care. Another thing that this application helped … and we expected … was data storage in a trusted way as we are moving from analogue to a digital world

[CHW, Nyarugenge District, FDG]

### Use and Perceived Benefits of the App

According to CHW reporting, the median number of people screened by each CHW ranged from 152 (IQR 70-276) for Nyarugenge to 24 (IQR 16-90) for Rusizi (Table 2). Of these, few were referred to a health center for diagnostic confirmation, between 1 and 5 per CHW. In total, 48.4% and 47.4% of the CHWs sent more than 5 people screened to the health centers in Gasabo and Kirehe, respectively. Positivity rates were higher in urban than rural districts. For example, more than half of the CHWs in Gasabo reported a confirmed positive case, whereas this rate was only 23.7% for Kirehe and 15.4% for Rusizi. Only 2 CHWs from Gasabo declared a case of death.

A high number of respondents considered that the most perceived benefit of using the e-ASCOV app was the enhancement of information sharing about COVID-19, followed by timely decision-making and quick dissemination of information.

The first thing, information got to be known so fast and this helps to make measures

[district director of health]

The way of using e-ASCOV or technology in delivering services and information about COVID-19 pandemic is used to know information about the pandemic earlier, so that the information can reach … the decision makers, and the patients may get treated in a quick way because technology might be quick than the ordinary ways in delivering information.

[CHW supervisor, central level]

Some of the respondents believed that using e-ASCov app would benefit quick data retrieval and decision-making. Respondents also shared that the e-ASCov app would benefit in avoiding COVID-19 infection spread by enhancing information storage because it was paperless and time-saving.

The benefits that I have seen in our application that we visited every household and asked the questions in the application and found that he was seriously sick at home but did not know the symptoms of COVID-19 and I immediately advised the client to go to the health center and accompanied the client and treat him right away; that is the benefit I have seen.

[CHW from Gasabo District]

**Table 2.** Description of overall intervention indicators according to the district.

Description		Gasabo (n=93, 24.6%)				Kirehe (n=97, 25.7%)				Nyarugenge (n=90, 23.8%)				Rusizi (n=98, 25.9%)				*P* value^a^
		n (%)	Median (IQR)			n (%)	Median (IQR)			n (%)	Median (IQR)			n (%)	Median (IQR)			
Total number of persons screened		93 (100)	86.0 (15.0-160.0)			97 (100)	48.0 (15.0-86.0)			90 (100)	152.5 (70.0-276.0)			98 (100)	24.0 (16.0-90.0)			<.001
Number of clients referred		93 (100)	5.0 (0.0-15.0)			97 (100)	5.0 (0.0-15.0)			90 (100)	2.0 (0.0-8.0)			98 (100)	1.0 (0.0-4.0)			.005
**Number of patients referred**																		<.001
	None	32 (34.4)	—^b^			27 (27.8)	—			35 (38.9)	—			44 (44.9)	—			
	1‐5	16 (17.2)	—			24 (24.7)	—			26 (28.9)	—			35 (35.7)	—			
	>5	45 (48.4)	—			46 (47.4)	—			29 (32.2)	—			19 (19.4)	—			
Number of clients confirmed positive among those referred		93 (100)	1.0 (0.0-4.0)			97 (100)	0.0 (0.0-0.0)			90 (100)	0.0 (0.0-2.0)			98 (100)	0.0 (0.0-0.0)			<.001
**At least 1 positive case**																		<.001
	No	42 (45.2)	—			74 (76.3)	—			55 (61.1)	—			83 (84.7)	—			
	Yes	51 (54.8)	—			23 (23.7)	—			35 (38.9)	—			15 (15.3)	—			
Number of confirmed cases with severe symptoms transferred for further management		93 (100)	0.0 (0.0-0.0)			97 (100)	0.0 (0.0-0.0)			90 (100)	0.0 (0.0-0.0)			98 (100)	0.0 (0.0-0.0)			.006
**At least 1 severe case**																		.006
	No	73 (78.5)	—			88 (90.7)	—			72 (80)	—			91 (92.9)	—			
	Yes	20 (21.5)	—			9 (9.3)	—			18 (20)	—			7 (7.1)	—			
Number of deaths among confirmed cases		93 (100)	0.0 (0.0-0.0)			97 (100)	0.0 (0.0-0.0)			90 (100)	0.0 (0.0-0.0)			98 (100)	0.0 (0.0-0.0)			.10
**At least 1 death**																		.10
	No	91 (97.8)	—			97 (100)	—			90 (100)	—			98 (100)	—			
	Yes	2 (2.2)	—			0 (0)	—			0 (0)	—			0 (0)	—			

a *P* value for *χ*2 test for qualitative variables or Student *t* test for quantitative variables.

b Not applicable.

### Satisfaction With and Challenges of the App

The satisfaction of CHWs is presented in Table 3. Overall satisfaction was higher in Nyarugenge (72.8/100) and Gasabo (80.7/100) than Kirehe (61.6/100) and Rusizi (64.5/100). More than 80% of CHWs were satisfied with the training offered before deployment of the app in all districts. In terms of availability of tools and internet connection, satisfaction was lower in rural than urban districts. More than 80% of the CHWs were willing to continue using the app, with the exception of CHWs in Kirehe (56.7%).

**Table 3.** Satisfaction (very satisfied/satisfied) of community health workers according to district.

		Gasabo (n=93; 24.6%)		Kirehe (n=97; 25.7%)		Nyarugenge (n=90; 23.8%)		Rusizi (n=98; 25.9%)		*P* value^a^
		n (%)	Mean (SD)	n (%)	Mean (SD)	n (%)	Mean (SD)	n (%)	Mean (SD)	
**Training provided in use of digital tool**										.23
	No	17 (18.3)	—^b^	8 (8.2)	—	11 (12.2)	—	13 (13.3)	—	
	Yes	76 (81.7)	—	89 (91.8)	—	79 (87.8)	—	85 (86.7)	—	
Score: 0‐100		93 (100)	81.7 (23.9)	97 (100)	85.1 (19.7)	90 (100)	80.8 (20.9)	98 (100)	79.1 (18.9)	.24
**Availability of equipment (telephone, etc)**										<.001
	No	27 (29.0)	—	76 (78.4)	—	19 (21.1)	—	47 (48.0)	—	
	Yes	66 (71.0)	—	21 (21.6)	—	71 (78.9)	—	51 (52)	—	
Score: 0‐100		93 (100)	81.2 (23.8)	97 (100)	54.4 (18.8)	90 (100)	70.8 (24.4)	98 (100)	57.9 (25.0)	<.001
**Internet connectivity**										<.001
	No	27 (29)	—	76 (78.4)	—	19 (21.1)	—	47 (48)	—	
	Yes	66 (71)	—	21 (21.6)	—	71 (78.9)	—	51 (52)	—	
Score: 0‐100		93 (100)	81.2 (23.8)	97 (100)	54.4 (18.8)	90 (100)	70.8 (24.4)	98 (100)	57.9 (25)	<.001
Simplicity/ease in use of the tool										<.001
	No	26 (28)	—	74 (76.3)	—	12 (13.3)	—	19 (19.4)	—	
	Yes	67 (72)	—	23 (23.7)	—	78 (86.7)	—	79 (80.6)	—	
Score: 0‐100		93 (100)	80.6 (23.9)	97 (100)	55.9 (20.7)	90 (100)	75.6 (23.1)	98 (100)	69.4 (27.2)	<.001
**Time used to fill and complete one record with the tool**										<.001
	No	27 (29)	—	76 (78.4)	—	19 (21.1)	—	47 (48)	—	
	Yes	66 (71)	—	21 (21.6)	—	71 (78.9)	—	51 (52)	—	
Score: 0‐100		93 (100)	81.2 (23.8)	97 (100)	54.4 (18.8)	90 (100)	70.8 (24.4)	98 (100)	57.9 (25)	<.001
**Getting technical assistance/support**										<.001
	No	42 (45.2)	—	76 (78.4)	—	33 (36.7)	—	52 (53.1)	—	
	Yes	51 (54.8)	—	21 (21.6)	—	57 (63.3)	—	46 (46.9)	—	
Score: 0‐100		93 (100)	69.9 (28.4)	97 (100)	55.2 (20.7)	90 (100)	59.2 (25.8)	98 (100)	49 (29.4)	<.001
**Delivery of services to the clients with the tool**										<.001
	No	26 (28)	—	74 (76.3)	—	12 (13.3)	—	19 (19.4)	—	
	Yes	67 (72)	—	23 (23.7)	—	78 (86.7)	—	79 (80.6)	—	
Score: 0‐100		93 (100)	80.6 (23.9)	97 (100)	55.9 (20.7)	90 (100)	75.6 (23.1)	98 (100)	69.4 (27.2)	<.001
**Continued use of the tool**										<.001
	No	15 (16.1)	—	42 (43.3)	—	11 (12.2)	—	17 (17.3)	—	
	Yes	78 (83.9)	—	55 (56.7)	—	79 (87.8)	—	81 (82.7)	—	
Score: 0‐100		93 (100)	89.2 (19.6)	97 (100)	77.3 (24.5)	90 (100)	78.6 (27.7)	98 (100)	75.3 29.9	<.001
Global satisfaction score: 0‐100		93 (100)	80.7 (19.4)	97 (100)	61.6 (15.4)	90 (100)	72.8 (19.2)	98 (100)	64.5 (21.6)	<.001

a *P* value of *χ*2 test for qualitative variables or Student *t* test for quantitative variables.

b Not applicable.

Most of the respondents stated that the main challenges were poor internet access or a poor network, CHWs having a low level of operational knowledge, lack of access to smartphones, and the shortage of equipment. Challenges were the lack of CHWs’ incentive and internet fees, use of a personal cell phone, low number of trained CHWs, limited access to electricity by CHWs, incompatibility of the app with CHWs’ cell phones, inability to use the app, inability to edit and correct an error, older CHWs feeling excluded, and the inability to retrieve information. The CHWs viewed the lack of smartphones as a factor that impeded reporting via the e-ASCov app. The poor internet connectivity was also highlighted by most of the CHWs as a limiting factor for better access.

## Discussion

### Principal Findings

This project confirmed the central position of CHWs in an effective strategy to deal with a pandemic such as COVID-19 in a pyramidal health system such as Rwanda’s. By including 2 districts, 2 rural and 2 urban, we were able to show that this type of system was possible in the health systems of countries where CHWs play a predominant role. Qualitative and quantitative results were consistent, and there was enthusiasm for the e-ASCov app. The smooth organization of the CHW network and its fully integrated nature into the Rwandan health system ensured that the app was easily deployed. In an epidemic context, CHWs were able to use this app as a screening and prevention tool to stem the spread of the epidemic in each district. The CHWs were able to use the app for screening suspected cases at the community level and were largely satisfied. However, there were some slight differences, particularly between urban and rural districts. Therefore, the conditions of implementation and transferability of such an app on a national scale need to be investigated in a broader context.

### Comparison With Prior Studies

This type of tool has been used in the African context for other diseases [10,11]. Although the use of the app was good in all districts, it was easier in urban than rural districts. This difference, beyond the organization of the CHW system in participating districts, seems to be linked to the level of education and CHWs’ experience in implementing this type of action. Indeed, in the urban districts, three-quarters of the CHWs had a level of education higher than primary school, whereas in the rural districts, the opposite was true. In addition, the smoothness of the internet connection in urban districts was also identified as a facilitating factor in the use of the app. Duclos et al [12] stated that mHealth implementation in rural health districts is expected to present considerable challenges, including technological barriers, organizational challenges, gender issues, confidentiality concerns, and unplanned aftereffects.

The successful implementation of the e-ASCov app at the community level opens up several possibilities. This type of app could be used in an epidemic context and in community-based monitoring of certain chronic diseases or maternal and child health [13]. The Rwanda CHW program was established in 1995. It aimed to strengthen essential maternal and child health care services through the education of pregnant women, health promotion and risk behavior reduction, follow-up, and linkages with health services. Rwanda has more than 50,000 CHWs (4 per village) with an organization of 3 CHWs (3 different dedicated functions) for each 100 to 150 households. One CHW is in charge of maternal and child health (identifying pregnant women, accompanying the regular monitoring of the pregnancy, and ensuring that the delivery takes place in a health facility). Two CHWs are responsible for referral and integrated community-based management of diseases (assessment, classification, and treatment) such as diarrheal diseases, pneumonia, malaria, and malnutrition in children younger than 5 years; provision of community-based contraceptives; directly observed treatment surveillance for tuberculosis; and prevention of noncommunicable diseases. The fourth CHW is specifically responsible for the hygiene of the village population. This organization allows for the use of connected tools for screening and monitoring several other diseases.

Although the implementation and experience of CHWs seem to support the good acceptability and feasibility of this type of tool, a positive perception of the population receiving the intervention is also important. This perception should be ensured by raising the awareness of the population, especially in rural areas, to improve their literacy in relation to digital health tools. For e-ASCov, part of the population was not sufficiently informed of the use of e-ASCov app. Those who were aware of it stated that the system provides useful information about COVID-19.

According to that technology of e-ASCOV, they did not use it. I think they were not even having it. I agree with what others have said...

[client, Kirehe District, FGD]

### Limitations

The first limitation is that the e-ASCov project was implemented in only 4 of the country’s 30 districts, which may restrict confidence in a full generalization of results. To minimize representation bias, we selected the 4 districts according to their rural or urban character and their geographical location. The 2 urban districts are located in the capital, where most of the cases of COVID-19 were identified (imported cases). The 2 other rural districts were located at the 2 extremities of the country: Rusizi in the western region bordering the Democratic Republic of Congo and Kirehe in the eastern region bordering Tanzania. Another limitation was the significant spread of the epidemic throughout the country, so the use of this type of app was less effective and less appropriate. Indeed, when the prevalence of the disease becomes very large and the strategy is to learn to live with the virus, community-based screening becomes less relevant. However, beyond COVID-19, the e-ASCov project reinforces the idea that digital tools such as smartphone apps can be of great use in community health. Despite these limitations, the e-ASCov project is one of the first projects to include more than 400 CHWs who were trained to use the app and who screened several thousand potential cases over a year. The results of this study have led the country’s health authorities to consider rolling out the app nationwide.

### Conclusion

Findings from this study show proof of concept that the use of mHealth tools by CHWs can enhance their participation and contribution to an effective and resilient health system in low- and middle-income countries. A study is needed of the generalization and transferability conditions of this type of app to other health conditions to enable CHWs to be given their full place in a pyramidal health system.

## Abbreviations

CHW: community health worker
FGD: focus group discussion
IDI: in-depth interview
mHealth: mobile health
